# Supplementary material for: Preventing postoperative pulmonary complications by establishing a machine-learning assisted approach (PEPPERMINT): Study protocol for the creation of a risk prediction model
Source: PLoS One. 2025 Aug 19;20(8):e0329076. doi: 10.1371/journal.pone.0329076 (PMC12364315; doi:10.1371/journal.pone.0329076)
Supplement: S1 File — (PDF) [file pone.0329076.s001.pdf]

Checklist PEPPERMINT Study

Part 1

1.	≥ 18 years old?	□ Yes	□ No
2.	Elective surgery?	□ Yes	□ No
3.	General anaesthesia planned?	□ Yes	□ No
4.	Postop. admission to normal ward/PACU planned?	□ Yes	□ No

Where all the questions in part 1 answered with „Yes“? Continue with **part 2** and **3**.

Part 2

1.	Age (in years):		□ ≤ 50	□ 51-80	□ ≥ 81
2.	Preoperative SpO_2 (in %):		□ ≥ 96	□ 91-95	□ ≤ 90
3.	Respiratory infection in last month?		□ Yes	□ No	
4.	Preop. anaemia (Hb ≤ 10g/dl) (History/Lab)?		□ Yes	□ No	
5.	Surgical incision:	□ Peripheral	□ Upper Abdominal	□ Intrathoracic	
6.	Planned duration of surgery (in h):		□ < 2	□ 2-3	□ > 3
7.	Emergency procedure?		□ Yes	□ No	

Summary informed consent:

Part 3

- Every **general anaesthetic** can lead to impairment of the lungs (**pulmonary complication**) due to certain risk factors.
- Often only oxygen administration is necessary, but severe complications with increased mortality are rare but possible
- The aim of this study is to **detect and treat possible pulmonary complications** after general anaesthesia at an early stage.
- Facts: **personal data on anaesthesia and surgery** (routinely collected without additional effort) as well as a **lung ultrasound** after the operation in the recovery room
- This ultrasound is performed while the patient is lying in bed and only takes about 5 minutes. It is a **painless examination with no side effects.**
- After the surgery, if you have not yet been discharged at these times, a **ward round** will be carried out on **days 1, 3 and 7** after the operation, during which we will ask you a few brief questions and listen to your lungs in order to identify any lung complications.
- When you are discharged from inpatient treatment, your participation in the study is complete.
- The PEPPERMINT study is an observational study, which means that participation in the study has **no effect on the course of your surgery, anaesthesia or post-operative course**.
