# Supplementary material for: Preventing postoperative pulmonary complications by establishing a machine-learning assisted approach (PEPPERMINT): Study protocol for the creation of a risk prediction model
Source: PLoS One. 2025 Aug 19;20(8):e0329076. doi: 10.1371/journal.pone.0329076 (PMC12364315; doi:10.1371/journal.pone.0329076)
Supplement: Attachment — Submitted filename: Response to Reviewers PEPPERMINT.pdf [file pone.0329076.s006.pdf]

PONE-D-24-22158
Preventing postoperative pulmonary complications by establishing a machine-learning assisted approach (PEPPERMINT): study protocol for the creation of a risk prediction model

Dear Sir or Madam,

Thank you very much for editing my manuscript. I look forward to the opportunity to improve my manuscript based on the reviewers' remarks. In the following, I will address all of the required concerns in a point-by-point answer.

Academic Editor:

1. *When submitting your revision, we need you to address these additional requirements. Please ensure that your manuscript meets PLOS ONE's style requirements, including those for file naming. The PLOS ONE style templates can be found at https://journals.plos.org/plosone/s/file?id=wjVg/PLOSOne_formatting_sample_main_body.pdf and https://journals.plos.org/plosone/s/file?id=ba62/PLOSOne_formatting_sample_title_authors_affiliations.pdf*

   I have checked the entire document and the attached files again for the formatting specifications and adjusted them.

2. *Thank you for stating the following in the Competing Interests section:*
   [I have read the journal's policy and the authors of this manuscript have the following competing interests: B. Jungwirth and S. Kagerbauer received grants from Löwenstein Medical Innovation (Berlin, Germany). M. Blobner received research support from MSD (Haar, Germany), fees for consultancy or lectures from GE Healthcare (Helsinki, Finland), Grünenthal (Aachen, Germany), and Senzime (Landshut, Germany).].
   Please confirm that this does not alter your adherence to all PLOS ONE policies on sharing data and materials, by including the following statement: ""This does not alter our adherence to PLOS ONE policies on sharing data and materials." (as detailed online in our guide for authors http://journals.plos.org/plosone/s/competing-interests). If there are restrictions on sharing of data and/or materials, please state these. Please note that we cannot proceed with consideration of your article until this information has been declared. Please include your updated Competing Interests statement in your cover letter; we will change the online submission form on your behalf.

   The above-mentioned fundings of the authors do not constitute a conflict of interest and therefore do not alter the adherence to PLOS ONE policies on sharing data and materials. I have added this statement to the manuscript (page 2, competing interests) and cover letter.

3. *In the online submission form, you indicated that [Due to German data protection regulations, we are not allowed to publish individual patient data. Access to the full protocol and data can be shared upon reasonable request from the corresponding*

author after appraisal by the data protection officer. The statistical code and machine learning code will be shared via a GitHub repository.]
All PLOS journals now require all data underlying the findings described in their manuscript to be freely available to other researchers, either 1. In a public repository, 2. Within the manuscript itself, or 3. Uploaded as supplementary information.
This policy applies to all data except where public deposition would breach compliance with the protocol approved by your research ethics board. If your data cannot be made publicly available for ethical or legal reasons (e.g., public availability would compromise patient privacy), please explain your reasons on resubmission and your exemption request will be escalated for approval.

We extended our statement in the section “Data availability” (page 3):

“Due to the sensitive nature of sensitive health data and in compliance with the General Data Protection Regulation (GDPR) as well as local ethics committee requirements, we are unable to publicly share the raw clinical and ultrasound data. However, to promote transparency and reproducibility, we will make the following resources available via a public repository (e.g., GitHub):

- The full source code and model architecture,
- A trained version of the predictive model,
- A fully anonymized test dataset for evaluation purposes,
- Detailed aggregated statistics describing the dataset, which will be provided in the supplementary material of the final publication.

Additionally, controlled access to the raw data can be granted after approval by the local data protection authority upon reasonable request in line with established data governance frameworks.”

Reviewer #1:

1. *The process of physicians deciding whether images meet criteria and potentially excluding patients introduces selection bias.*

We thank the reviewer for this valuable remark.

To ensure a minimum diagnostic standard for lung ultrasound quality, the following inclusion criteria for image assessment were defined and stated on page 12: “At least two adjacent ribs and the pleura or corresponding pathologies (e.g., pneumothorax, pleural effusion) must be visible on the ultrasound image. The ultrasound examination must cover all 12 defined areas.”
On page 15 we added the sentence: “If these conditions are not met, the interpretability of the ultrasound is considered insufficient.”

We added a further description of the ultrasound technique in the section “Study measures” on page 15:” To maximize inclusiveness and minimize selection bias, operators are encouraged to adjust technical parameters (e.g., depth and gain) during image acquisition in order to meet these quality criteria. A convex transducer was selected to allow visualization of deeper structures across a wide range of patient anatomies.”

With regard to the exclusion of images, we have added the following note on page 12 to the “Eligibility criteria” section: “Importantly, decisions regarding potential exclusion due to poor image quality will not be made by the examining physician, but rather by an independent, blinded radiologist, thereby minimizing the risk of subjective selection bias.”

2. *Using mean or modal imputation for missing data is overly simplistic and can introduce bias. More sophisticated methods like multiple imputation may be considered.*

We thank the reviewer for addressing this important point and have added some notes to clarify the procedure regarding data imputation. Therefore, we have re-written the section “Statistical methods” and added a subsection “Missing Values and Data Imputation” on pages 29 and 30.
“With regard to imaging data, only complete data sets will be accepted for analysis. Missing values in the clinical dataset will initially be handled using the default preprocessing pipeline in the case that H2O AutoML is used. This pipeline applies median or mean imputation for numerical variables and mode imputation for categorical variables (https://docs.h2o.ai/h2o/latest-stable/h2o-docs/data-munging/imputing-data.html). In addition to this standard approach, we will evaluate alternative imputation strategies, particularly for approximately normally distributed numerical variables. These include, e.g., k-nearest neighbors (kNN) imputation, which leverages similarities across observations in the multivariate feature space and multiple imputation using chained equations (MICE), which incorporates multivariable relationships and reflects the uncertainty associated with imputed values.
Given the prospective nature of the study, we anticipate that most missing data will not be missing at random (NMAR), but rather occur systematically, for instance, due to early patient discharge or an inability to perform assessments as a result of clinical deterioration. In such cases, the absence of data may itself be informative with respect to the patient’s risk for postoperative complications. Therefore, in addition to the imputation techniques mentioned above, we will explore modelling strategies that explicitly account for informative missingness. These include the incorporation of missingness indicators (binary flags denoting whether a variable was observed or missing) and sensitivity analyses to assess the robustness of model predictions under different assumptions about the missing data mechanism.
The final approach to handling missing data will be selected based on a combination of model performance, diagnostic checks, and clinical plausibility.”

3. *What test was used for the sample size calculation?*

Sample size estimation was based on the expected improvement in the area under the receiver operating characteristic curve (AUROC) compared to the established ARISCAT score (AUROC ≈ 0.83). Assuming an anticipated AUROC of 0.93 for our model, the required number of cases was calculated using the method described by Hanley and McNeil for comparing ROC curves. We added this important detail in the section “Sample size” on page 17: “With a significance level of 0.05 and a power of 80%, 512 patients would be needed to create the database based on the method described by Hanley and McNeil for comparing ROC curves (Hanley JA, McNeil BJ. A method of comparing the areas under receiver operating characteristic curves derived from the same cases. Radiology. 1983;148(3):839-43.)

4. *While AUROC is mentioned, in imbalanced datasets (which are common in medical studies), AUPRC may be more informative.*

We agree with the reviewer that AUROC is of limited use for assessing model quality in unbalanced data sets. However, the current literature on the ARISCAT score only gives values for the AUROC and not for the area under the precision-recall curve (AUPRC). Our sample size calculation is therefore based on the AUROC. We added this aspect in the section “Sample size” on page 17: “It should be noted that the current literature does not provide any further metrics such as area under the precision-recall curve (AUPRC) or F1 score on the ARISCAT (15,16). We therefore used the AUROC as the reference metric.”

The final model in our study will be, of course, measured against other metrics such as the AUPRC and the F1 score in order to adequately evaluate model performance on an unbalanced data set as described in the revised subsection “Model Training and Evaluation” of the “Statistical Methods” section on page 27: “Model performance will be evaluated on the independent hold-out test set using several complementary performance metrics. These will include the area under the receiver operating characteristic curve (AUROC), the area under the precision-recall curve (AUPRC), overall accuracy, sensitivity, specificity, F1-score, as well as positive and negative predictive values.”

5. *The use of complex deep learning models with relatively limited data raises concerns about overfitting, especially with no clear regularization strategy mentioned.*

We thank the reviewer for this important comment. Given the limited sample size, we acknowledge the risk of model overfitting. To mitigate this, we will implement a range of regularization strategies commonly used in deep learning and have included this in the revised version of our manuscript in the section “statistical methods” where we have added a subsection “Addressing Overfitting and Class Imbalance” on page 28 and 29:

“Overfitting will be addressed through the implementation of several regularization techniques commonly used in deep learning. These include dropout layers within the network architecture, L2 weight regularization to penalize large weights, and data augmentation techniques applied to ultrasound images (e.g., rotation, flipping, and zooming). Model complexity will also be restricted as needed.”

6. *There are no plans for cross-validation or external validation.*

We thank the reviewer for highlighting the importance of model validation. We would like to clarify that k-fold cross-validation will be used during model training to ensure internal validity and to minimize overfitting. This is now clearly described in the revised version of the study protocol. We have added this aspect in the subsection “Model Training and Evaluation” of the “Statistical methods” section on page 27 and 28:
“The primary model development will be based on a dataset of 512 patients. Within this cohort, we will implement stratified k-fold cross-validation (typically 5- or 10-fold, depending on the final class distribution) where possible to assess model

robustness and mitigate overfitting. Image-based deep learning models require extensive computational resources, therefore, cross-validation will be applied more selectively. We will therefore use a fixed training/validation/test split for imaging data, ensuring non-overlapping patient groups. Early model experiments will be conducted using internal validation on the training data with hold-out validation to optimize architecture and hyperparameters. Final model evaluation will be performed on an independent hold-out test set."

Furthermore, we highlight our plans for external validation in the subsection "External and Subgroup Validation" on pages 30 and 31:

"While model development and internal validation will be performed using data from a single clinical centre, further validation is planned at a second site of our hospital that predominantly treats gynaecological and ENT patients. This cohort will serve as a distinct population for assessing model generalizability across surgical disciplines. Additionally, the model will be evaluated in a separate high-risk subgroup comprising patients undergoing urgent or emergency procedures, such as elderly individuals with hip fractures. These patients were intentionally excluded from the initial development cohort and will offer insight into the model's performance in more acute care settings."

7. *Given that POPC is likely a relatively rare event, there's no clear strategy for dealing with potential class imbalance in the dataset.*

We acknowledge the reviewer's concern regarding potential class imbalance and have conducted a small observational study in advance. We have added the results of this observation and potential strategies for addressing class imbalance in the subsection "Addressing Overfitting and Class Imbalance" on page 28 and 29:

" To better estimate the prevalence and clinical relevance of postoperative pulmonary complications (POPC), we conducted a preliminary single-centre observational study involving 259 patients undergoing surgery under general anaesthesia. The cohort included 106 female patients (41%) and 62 current smokers (24%) with a median age of 66 years. Overall, 111 patients (43%) experienced at least one POPC, indicating that POPC is a relatively frequent complication in the targeted patient population. Based on this preliminary observational data, only moderate class imbalance is to be expected. Nevertheless, additional strategies will be employed to ensure balanced learning. These include maintaining class ratios across cross-validation folds, monitoring class-wise performance metrics, and using class weighting techniques if imbalances are observed in specific subgroups."

8. *Using Youden's index for cut-off determination may not be optimal, especially if the costs of false positives and false negatives are not equal in this clinical context. How will the risk score be calculated?*

We thank the reviewer for this important observation. We agree that relying solely on Youden's index may not reflect clinical priorities, especially if the consequences of false positives and false negatives differ. We now take this aspect into account in the revised version of the manuscript and have added a paragraph in the subsection "Model Training and Evaluation" on page 27 and 28:

"Importantly, our models are designed to output individualized risk probabilities, not dichotomous predictions. As such, the selection of a cut-off point for potential clinical decision-making will be a post-modelling step, and risk thresholds for high-risk patient

classification will not be predefined. Instead, various thresholding strategies will be explored post hoc, including Youden's index (for balanced sensitivity and specificity), cut-offs optimized for specific clinical priorities (e.g., maximizing sensitivity), and thresholds guided by DCA."

Reviewer #2:

1. *The core element in developing a disease risk prediction model using AI and machine learning is the algorithm itself. Developing such a model requires expertise in machine learning. Please provide more details about the professionals involved in your team, the design of the model, and the software tools utilized.*

   We agree with the reviewer that professional qualification is an indispensable prerequisite for the development of a clinically applicable model. Development and implementation of the predictive model will be carried out by an interdisciplinary team with strong expertise in both clinical medicine and machine learning. We have now gone into this fact in more detail in the "Study setting" section on pages 10 and 11: "The members of the study group are predominantly physicians and specialists in the fields of anesthesiology and radiology. The leader of the research group "Experimental Radiology", who holds a formal background in engineering, is part of the study team. His expertise lies in machine learning, deep learning, and computer vision, with a focus on medical imaging applications. Another group member holds a master's degree in medical informatics and has substantial experience in machine learning for perioperative risk prediction. The study team is completed by Master's and PhD students from the Department of Radiology with a background in computer science and engineering"

   In the "Statistical methods" section, we have added a subsection "Overview of Modelling approaches", where we provide more details about model design and software tools on page 26 and 27:
   "Three predictive modelling strategies will be pursued. First, an imaging model will be created using deep learning techniques applied to lung ultrasound images. This model will be developed in Python using the PyTorch framework. Transfer learning will be employed, utilizing pretrained convolutional neural network (CNN) architectures such as ResNet or DenseNet. These models will be fine-tuned on the study-specific dataset. We will also assess the performance of medical foundation models and contrastive learning-based pretraining to optimize feature extraction from the ultrasound images. Second, a clinical model will be developed using only structured patient data. This model will be trained using frameworks like H2O AutoML within R/RStudio as well as built-in R functions, which allows for the systematic evaluation and tuning of various machine learning algorithms, including gradient boosting machines, random forests, and neural networks.
   Third, a combined model will integrate both clinical and imaging data. Various fusion strategies will be explored, including early and late fusion, to determine the most effective method for combining heterogeneous data types. The final architecture for this combined model will be selected based on performance observed in a hold-out test dataset."

2. *Has the model been externally validated? How do you plan to ensure its broad applicability across diverse clinical settings?*

We thank the reviewer for addressing this important point, which was also criticized by Reviewer 1. We would therefore like to refer you to the reply to Reviewer 1, point 6. In short, we are using cross-validation and are also planning validation at a second hospital site and on a different patient population.
We have included this aspect in the "Statistical methods" section. Cross-validation is described on page 27 of the revised study protocol, external validation on pages 30 and 31.

3. *Lung ultrasound has limited use in clinical practice due to the interference of air in the lungs. Additionally, the complexity of the ultrasound images is influenced by factors such as probe position and angle. How to ensure the accuracy and reliability of the model considering these challenges?*

   We agree with the reviewer that examiner dependence is a frequently expressed concern in the setting of ultrasound examinations for research purposes. We have taken various measures to address this issue:
   - We have chosen a 12-zone model that has the highest intra-class correlation coefficient. We have included this on page 14 under "study measures": "This 12-zone protocol showed the highest intra-class correlation coefficient compared to other protocols (14)."
   - We defined inclusion criteria to ensure a minimum diagnostic standard for lung ultrasound quality which we highlight on page 12 in the section "eligibility criteria": "Ultrasound images on which the leading structures ribs and pleura or alternative pathologies cannot be depicted, or incomplete visualisation of the 12 previously defined examination areas in the thoracic region, e.g., in the case of immobile patients or inaccessibility due to a bandage or drains."
   - Exclusion due to poor image quality will not be decided by the performing anesthesiologist, but by a qualified radiologist. This is stated on page 12: "Importantly, decisions regarding potential exclusion due to poor image quality will not be made by the examining physician, but rather by an independent, blinded radiologist, thereby minimizing the risk of subjective selection bias."
   - A convex transducer is specified in the standard protocol on page 15: "A convex transducer was selected to allow visualization of deeper structures across a wide range of patient anatomies."

4. *Pulmonary complications can be presented in a variety of ways, with differing severity. Consequently, there will likely be numerous outcome events (endpoints) in this study. Some mild postoperative pulmonary complications may not require intervention, and predicting such cases may not provide substantial clinical value. Does this study aim to focus specifically on identifying the more severe pulmonary?*

   We thank the reviewer for highlighting the clinical relevance of differentiating between mild and severe postoperative pulmonary complications (POPC). In our study, the type and severity of POPC will be classified according to the internationally recognized StEP (Standardized Endpoints in Perioperative Medicine) criteria, which include atelectasis, pneumonia, ARDS, pulmonary aspiration, respiratory failure, pulmonary embolism, pleural effusion, pneumothorax, bronchospasm, and cardiogenic pulmonary edema as described in table 2. The primary model will be a binary classification model predicting the endpoint POPC regardless of severity. However, secondary models will be provided to take into account the type and severity of the POPC. We have specified this goal in the "Secondary Models and Endpoints" section

on page 30:

“In addition to the primary binary classification model for the presence or absence of POPC, the study will explore the development of additional models that aim to distinguish between different types and severities of complications. These extended models will incorporate more granular outcome definitions, including the timing of complication onset and the presence of multiple complications in a single patient.

Moreover, secondary predictive models will be constructed to estimate outcomes such as quality of postoperative recovery, length of hospital stay, and in-hospital mortality.”

I am looking forward to your answer.

Kind regards,

Dr. med. Britta Trautwein
(Corresponding author)
